# Association between increased BMI and cognitive function in first-episode drug-naïve male schizophrenia

**DOI:** 10.3389/fpsyt.2024.1362674

**Published:** 2024-03-05

**Authors:** Xing Deng, Shuiping Lu, Yan Li, Xinyu Fang, Rongrong Zhang, Xuran Shen, Jinglun Du, Shiping Xie

**Affiliations:** Department of Psychiatry, The Affiliated Brain Hospital of Nanjing Medical University, Nanjing, China

**Keywords:** schizophrenia, cognitive function, BMI, overweight/obesity, normal/under weight

## Abstract

**Objective:**

Although the adverse effects of obesity in schizophrenia are documented, there is limited research exists on the implications for untreated initial schizophrenia. Our investigation aimed to explore the connections between BMI and cognitive function in first-episode drug-naïve (FEDN)schizophrenia.

**Methods:**

We enrolled 143 FEDN schizophrenia patients, and collected data on their body mass index, fasting blood glucose and lipid levels. Cognitive function was measured with the MATRICS Consensus Cognitive Battery (MCCB). Using correlation and regression analysis to assess the relationship between BMI and cognitive performance.

**Results:**

The prevalence rate of overweight plus obesity in FEDN schizophrenia patients was 33.57%. Patients with FEDN schizophrenia exhibited extensive cognitive impairment, and those who were overweight/obesity demonstrated more severe impairments in working memory and visual learning when compared to normal/under weight counterparts. Correlation analysis indicated a negative association between working memory and BMI and TG, as well as a link between visual learning and BMI and LDL-C. Multiple linear regression analysis revealed that a higher BMI predicted a decrease in working memory in FEDN schizophrenia patients.

**Conclusion:**

Our results indicate that the rate of overweight plus obesity is high in FEDN schizophrenia patients, and there is an association between BMI and cognitive function in schizophrenia, particularly in relation to working memory.

## Introduction

Schizophrenia is a chronic disease with an unclear etiopathogenesis and poor prognosis, representing a heavy economic burden (1). According to epidemiological studies, up to 50% of patients with schizophrenia experience obesity problems (2), and approximately 44% of untreated first-episode schizophrenia patients are overweight or obese (3), which is higher than the rate among general population (2, 3). Furthermore, obesity in individuals with schizophrenia not only increases their vulnerability to physical ailments like cardiovascular disease but also diminishes their life expectancy by approximately a decade (4, 5).

Cognitive impairment is a significant and prevalent issue in schizophrenia (6). A number of studies consistently underscored that cognitive impairment hinders the recovery of social functioning in schizophrenia and is the most challenging core symptom to significantly improve (7–9). Previous research has discovered a connection between obesity and cognitive dysfunction. For instance, obese mice displayed poorer learning and memory capabilities than non-obese counterparts (10, 11). A recent study found that schizophrenia patients had lower working memory, motor speed, and cognitive composite scores as BMI increased (12). Another study pointed out that elevated BMI may detrimentally affect neurocognitive function by disrupting white matter integrity in schizophrenia (13). However, C. W. Wei et al. discovered a positive correlation between BMI and verbal and visuospatial abilities in schizophrenia (14). Furthermore, Rashid et al. suggested that obesity does not exert a direct impact on cognitive function in schizophrenia (15).

However, the aforementioned studies primarily focused on chronic schizophrenia patients, and the results could be influenced by prolonged psychiatric symptoms and the administration of antipsychotic medications (16–18). Antipsychotic medications, especially atypical antipsychotics, have been indisputably linked to obesity-related metabolic disturbances (17, 18). There is also intense debate regarding the impact of antipsychotic drugs on cognitive function (19). Furthermore, there are gender differences in the psychiatric symptoms and cognitive functions of patients with schizophrenia (20). Similarly, Zhu Y et al. found that attentional bias during social information processing also exhibits gender differences (21). There is little research examining the association between obesity and cognition in first-episode drug-naïve (FEDN) schizophrenia patients. Therefore, the insufficient evidence regarding the cognitive implications of obesity in schizophrenia calls for further investigation to establish a clearer understanding.

Considering the sex differences in schizophrenia (20, 21) and the effect of antipsychotic medication (16, 17, 22), we recruited first-episode drug-naïve male patients with schizophrenia to control for these confounding factors. We collected metabolic indicators, including BMI, fasting plasma glucose, and lipids, as well as symptom dimensions and cognitive assessment scores. The primary objective of this study was to investigate the differences in cognitive function between overweight/obesity and normal/under weight FEDN male patients with schizophrenia, followed by an investigation into the correlation between BMI and cognitive function. We postulated that a significant correlation would exist between BMI and cognitive ability in FEDN male schizophrenia. Building upon prior research, we recruited drug-naïve male patients with first-episode schizophrenia to control for these confounding factors.

## Materials and methods

### Participants

Our study subjects were 143 patients enrolled in the outpatient clinic and inpatient departments of the Affiliated Brain Hospital of Nanjing Medical University from May 2017 to October 2022. All patients were unanimously assessed by two highly experienced associate chief psychiatrists or chief psychiatrists, in accordance with the diagnostic criteria outlined in the Diagnostic and Statistical Manual of Mental Disorders (DSM)-5. All study participants were diagnosed with schizophrenia after at least 1 year of follow-up. The inclusion criteria for schizophrenic patients were as follows: (1) the Chinese Han population, right-handed, aged 16-44; (2) Education years ≥ 8 years, intelligence quotient (IQ) ≥ 70; (3) First onset, duration of first psychotic symptoms ≤ 24 months, drug naïve (i.e., no previous exposure to antipsychotics), no prior exposure to physical therapy; (4) Positive and Negative Syndrome Scale (PANSS) total score ≥ 60 points. Exclusion criteria included major somatic disorders, organic mental disorder, dementia/mental retardation, alcohol or substance abuse. Participants provided written consent, and the Medical Research Ethics Committee of the Affiliated Brain Hospital of Nanjing Medical University approved this consent procedure.

### Clinical and psychological assessments

Age, sex, and years of education were self-reported. Height and weight were measured, and body mass index (BMI) was calculated using the formula BMI = weight (kg)/height squared (m^2^). Based on the Chinese metabolic abnormality criteria (23), individuals with a BMI≥24 kg/m^2^ were considered overweight/obesity group, while those with a BMI < 24 kg/m^2^ were classified as normal/under weight group.

Fasting blood samples were collected between 6:30-7:30 in the morning. The levels of fasting blood glucose (FBG), total cholesterol (TC), triglyceride (TG), low-density lipoprotein cholesterol (LDL-C), and high-density lipoprotein cholesterol (HDL-C) were measured using Beckman AU5821 automatic biochemical analyzer.

We computed duration of illness (DUI) from onset of illness to the date of assessment. Intellectual quotient (IQ) was measured using the Chinese version of the Wechsler Adult Intelligence Scale-Revised (WAIS), which includes four subtests: Knowledge Quiz, Similarity Test, Picture Filling Test, and Block Diagram Test. Psychopathology was assessed using the Positive and Negative Syndrome Scale (PANSS), which was administered by two experienced psychiatrists who received an intra-class correlation coefficient (ICC) above 0.8 prior to the study inception. All data collection was completed in 5 days.

### Measures of cognitive function

The cognitive functions were assessed using the Chinese version of the MATRICS Consensus Cognitive Battery (MCCB) (24). MCCB included 9 sub-items, which were Trail Making Test, Symbol Coding, Hopkins Verbal Learning-Revised, Spatial Span, Mazes, Brief Visuospatial Memory Test-Revised, Fluency, Managing Emotions, and Continuous Performance Test-Identical Pairs. Standardized T scores were calculated for each subtest to assess composite scores and the following seven cognitive domains: speed of processing, attention and vigilance, working memory, visual learning, verbal learning, problem solving, and social cognition (25).

### Statistical analyzes

The analysis was conducted using SPSS 27.0. The normality of the data distribution was assessed using the Shapiro-Wilk test. After conducting this test, it was found that all quantitative data adhered to a normal distribution. Independent samples t-test was employed to compare the demographic, clinical characteristics, and cognitive function between groups. The cognitive function of norm and patients was compared using one sample t-test. Pearson correlation analysis was used to explore preliminary associations between BMI and cognitive function. Multiple linear regression analysis was employed to further explore the relationship. In this analysis, cognitive function served as the dependent variable, while meaningful indicators identified through correlation analysis were considered independent variables. Additionally, we controlled for covariates such as age, IQ, and DUI.

## Results

### Demographic and clinical characteristics

The demographic, metabolic characteristics and PANSS scores are shown in **Table 1**. The rate of overweight/obesity among FEDN patients with schizophrenia was 33.57% (48/143), which was higher than that of the general population aged 25-33 years (19.10%) (26). Compared to normal/under weight patients, overweight/obesity patients showed significantly elevated levels of BMI, TG and LDL-C (all *p <*0.05). However, HDL-C is lower in the overweight/obesity group than in the normal/under weight group (*p <*0.05). There were no significant differences in the remaining demographic variables and PANSS scores between the two groups.

**Table 1:** Demographic, metabolic characteristics and PANSS scores of first-episode drug-naïve schizophrenia patients.

	Schizophrenia (n = 143)	groups		t	*p*
		normal/under weight(n = 95)	overweight/obesity (n = 48)		
Age (years)	25.73 ± 7.31	24.94 ± 7.05	27.31 ± 7.64	t = -1.850	0.066
Years of Education	13.07 ± 2.72	12.89 ± 2.93	13.42 ± 2.26	t = -1.178	0.241
IQ	107.90 ± 11.70	107.92 ± 11.83	107.86 ± 11.58	t = 0.029	0.977
DUI (months)	11.29 ± 8.18	10.93 ± 8.10	12.00 ± 8.38	t = -0.740	0.461
BMI (kg/m^2^)	22.75 ± 4.01	20.42 ± 1.96	27.35 ± 2.87	t = -17.010	<0.001***
FBG (mmol/L)	4.51 ± 0.56	4.51 ± 0.57	4.51 ± 0.55	t = 0.017	0.986
TG (mmol/L)	1.15 ± 0.79	0.98 ± 0.61	1.50 ± 1.00	t = -3.292	0.002**
TC (mmol/L)	4.06 ± 0.88	3.97 ± 0.74	4.23 ± 1.09	t = -1.490	0.141
LDL-C (mmol/L)	2.29 ± 0.74	2.17 ± 0.63	2.51 ± 0.88	t = -2.388	0.020*
HDL-C (mmol/L)	1.15 ± 0.25	1.23 ± 0.23	1.00 ± 0.22	t = 5.660	<0.001***
PANSS					
Positive symptom	23.76 ± 3.59	23.48 ± 3.75	24.29 ± 3.22	t = -1.274	0.205
Negative symptom	19.35 ± 3.85	19.36 ± 3.95	19.33 ± 3.67	t = 0.036	0.971
General psychopathology	45.24 ± 3.44	45.01 ± 3.38	45.69 ± 3.54	t = -1.112	0.268
Total symptom	88.30 ± 7.25	87.85 ± 7.28	89.19 ± 7.170	t = -1.041	0.300

Means ± SD value, *p < 0.05, **p < 0.01, ***p < 0.001.

IQ, intelligence quotient; DUI, duration of illness; BMI, body mass index; FBG, fasting blood glucose; TC, total cholesterol; TG, triglyceride; LDL-C, low-density lipoprotein cholesterol; HDL-C, high-density lipoprotein cholesterol; PANSS, Positive and Negative Syndrome Scale.

### Cognitive function

**Figure 1:**
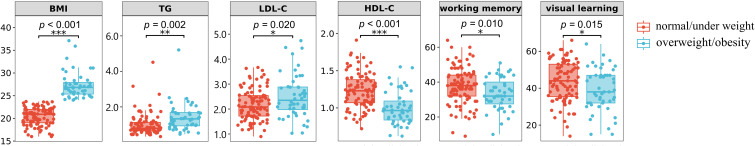
Box plots of significant differences between normal/under weight and overweight/obesity schizophrenia.

FEDN schizophrenia patients exhibited significant cognitive deficits compared to Chinese MCCB norms (n = 656, mean = 50, SD = 10) (24), as stated in **Table 2** (all *p* < 0.001). And the cognitive differences between overweight/obesity and normal/under weight FEDN schizophrenia patients were demonstrated in **Table 3**. overweight/obesity patients exhibited markedly inferior performance in working memory and visual learning compared to normal/under weight patients. Box plots depicting significant differences between the two groups are presented in **Figure 1**. However, no significant differences were observed between the two groups in the remaining cognitive domains.

**Table 2:** Cognitive function in FEDN schizophrenia patients and controls.

	N	Mean	SD	Controls Mean	t	95% CI	*P*
speed of processing	143	39.56	10.94	50.00	-11.417	-12.25, -8.63	<0.001***
attention and vigilance	143	37.42	10.29	50.00	-14.617	-14.28, -10.88	<0.001***
working memory	143	36.43	10.24	50.00	-15.844	-15.27, -11.88	<0.001***
verbal learning	143	38.57	11.57	50.00	-11.807	-13.34, -9.51	<0.001***
visual learning	143	42.20	11.26	50.00	-8.286	-9.67, -5.94	<0.001***
problem solving	143	43.92	11.01	50.00	-6.607	-7.90, -4.26	<0.001***
social cognition	143	35.34	10.18	50.00	-17.220	-16.34, -12.97	<0.001***
composite scores	143	32.29	11.23	50.00	-18.863	-19.57, -15.86	<0.001***

CI, confidence interval.

***p < 0.001.

**Table 3:** Cognitive function in normal/under weight and overweight/obesity schizophrenia patients.

	normal/under weight (n = 95)	overweight/obesity (n = 48)	t	*p*
Speed Of Processing	39.60 ± 10.75	39.48 ± 11.41	t = 0.062	0.951
Attention And Vigilance	38.02 ± 10.16	36.23 ± 10.55	t = 0.983	0.327
Working Memory	37.98 ± 10.52	33.35 ± 9.02	t = 2.600	0.010*
Verbal Learning	38.54 ± 11.39	38.65 ± 12.04	t = -0.053	0.958
Visual Learning	43.81 ± 10.84	39.00 ± 11.50	t = 2.454	0.015*
Problem Solving	43.87 ± 10.84	44.00 ± 11.47	t = -0.065	0.949
Social Cognition	35.61 ± 10.38	34.81 ± 9.85	t = 0.441	0.660
Composite Scores	33.22 ± 10.94	30.44 ± 11.67	t = 1.405	0.162

*p < 0.05.

### Association between BMI and cognitive function

**Figure 2:**
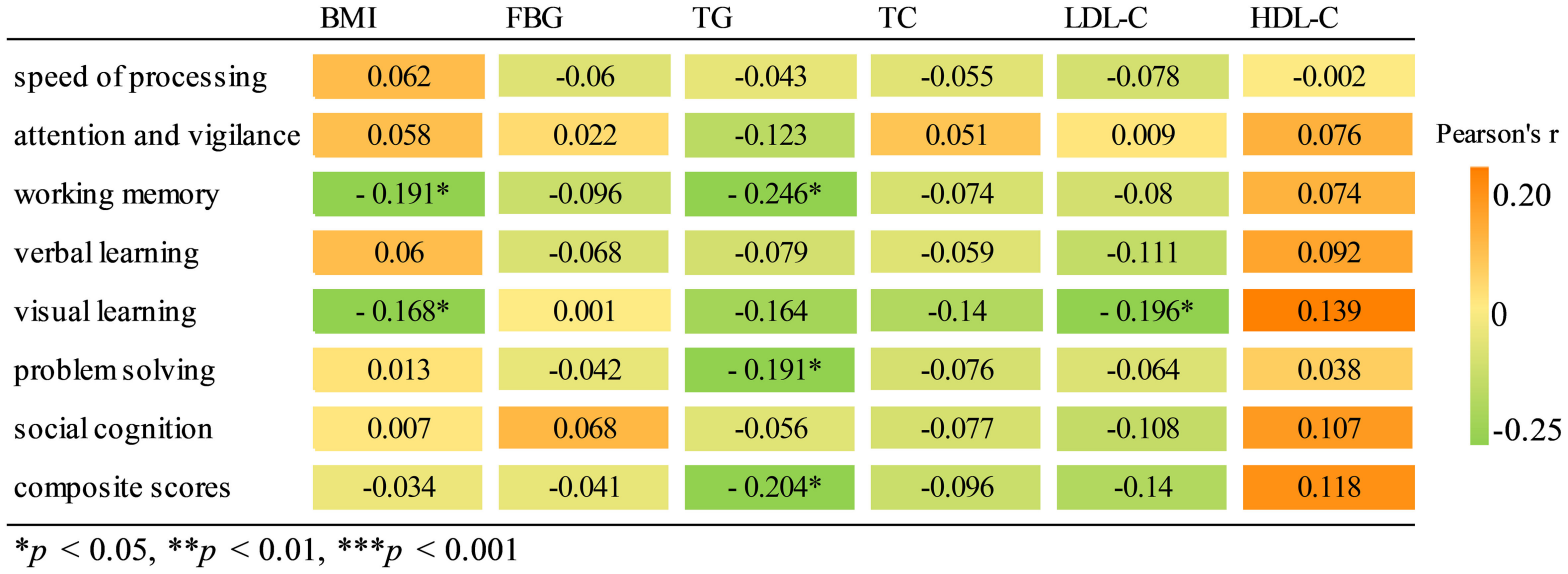
Pearson correlation heatmap among cognitive function and metabolic parameters.

Pearson correlation analysis revealed a significant negative association between working memory and BMI (r = -0.191, *p* = 0.022) as well as TG (r = -0.246, *p* = 0.003). Similarly, visual learning exhibited a significant negative correlation with both BMI (r = -0.168, *p* = 0.045) and LDL-C levels (r = -0196, *p* = 0.019). There was a negative correlation between TG and problem solving (r = -0191, *p* = 0.023), as well as composite scores (r = -0204, *p* = 0.014). Results of correlation analysis are shown in a heatmap in **Figure 2**. The scatter plot of BMI and cognitive function are shown in **Supplementary Figure S2**. Further multiple regression analysis revealed that only a higher BMI predicted a decrease in working memory (β = -0.439, *p* = 0.044, **Table 4**).

**Table 4:** Results of the regression analysis.

	BMI	TG	LDL-C	Age	IQ	DUI	R^2^
working memory	-0.439*	-2.185	-	0.166	0.209**	-0.095	0.142
visual learning	-0.399	-	-1.963	0.037	0.337***	-0.133	0.193

*p < 0.05, **p < 0.01, ***p < 0.001.

BMI, body mass index; TG, triglyceride; LDL-C, low-density lipoprotein cholesterol; IQ, intelligence quotient; DUI, duration of illness.

## Discussion

The primary findings of this study were as follows: (1) the rate of overweight/obesity among FEDN schizophrenia patients was 33.57%. (2) FEDN schizophrenia patients had extensive cognitive impairment. And overweight/obesity patients exhibited markedly inferior performance in working memory and visual learning compared to normal/under weight patients. (3) Correlation analysis indicated a significant association between lower working memory and higher BMI and TG, as well as a link between poorer visual learning and elevated BMI and LDL-C in FEDN schizophrenia patients. Further regression analysis revealed that BMI significantly and negatively influenced working memory among FEDN schizophrenia patients.

In this study, we observed a higher overweight/obesity rate (33.57%) among FEDN schizophrenia patients (age 25.73 ± 7.31) compared to the general population aged 15-24 years old (7.50%) and 25-33 years old (19.10%) (26). Our findings are consistent with the previous study by Y. Tian et al., which reported an increased prevalence of overweight and obesity among first-episode schizophrenia in contrast to healthy control (3). Moreover, metabolic syndrome is more likely to develop in both drug-naïve schizophrenia patients and their siblings, regardless of antipsychotic effects (27). The findings suggest a greater propensity for overweight and obesity among individuals with schizophrenia, independent of antipsychotic medication impact.

The increased prevalence of overweight/obesity in first-episode schizophrenia could be attributed to a combination of factors, including unhealthy lifestyles, gut microbiota dysbiosis, and genetic susceptibility. Unhealthy lifestyles, such as reduced time spent on complex activities (28) and excessive consumption of nonalcoholic beverages (i.e., high sugar intake) (29), are common among patients with early-stage schizophrenia and contribute to the elevated obesity rate in this population. In addition, dysbiosis of the gut microbiota may provide a common biological basis for the etiology of schizophrenia and obesity. Both conditions are associated with a reduction in anti-inflammatory bacteria and an increase in proinflammatory and pathogenic bacteria (30). The inflammatory signals produced by gut microbiota may serve as a potential link between schizophrenia and obesity.

More interestingly, several studies suggest that the higher obesity rate among first-episode schizophrenia patients may be attributed to genetic susceptibility. The 22q11.2 deletion syndrome, for instance, is the strongest risk factor for schizophrenia (31, 32) and is also associated with a higher risk of developing obesity (33, 34). A study found a strong correlation between schizophrenia and BMI at 18 different genetic loci, with 16q12.11 being the primary locus (35). The duplication or deletion of 16p11.2 can cause various physical and mental symptoms, including schizophrenia and obesity (36–39). The shared genetic loci between BMI and schizophrenia are linked to 20 significantly enriched pathways, with the proton pump inhibitor pathway and AKT phosphorylation targets in the nuclear pathway being particularly significant (40). These pathways have an impact on neurodevelopment (40), providing insights into the biological mechanisms underlying the relationship between schizophrenia and obesity in terms of genetic susceptibility.

In this study, our second finding is that FEDN schizophrenia patients had significantly lower cognitive function than the norm across multiple domains. This is consistent with previous research (41–43), which demonstrated that early-stage schizophrenia patients suffer from widespread cognitive impairment. Moreover, overweight/obesity schizophrenia patients performed significantly worse than normal/under weight patients in working memory and visual learning. This phenomenon may be related to the detrimental effects of obesity on cognitive function, which has also been corroborated by prior studies (10–13).

However, the exact mechanism underlying the association between obesity and cognitive impairments in schizophrenia is complex and not fully understood. Obesity may lead to inflammation, insulin resistance, vascular changes, and alterations in brain structure and function. It can trigger inflammation pathways in microglial cells, causing abnormalities in cerebral blood vessels and potentially leading to cognitive decline (44, 45). Obesity-related insulin resistance can disrupt gene expression in the hippocampus, impairing cognitive functions (46). Obesity is also associated with increased amyloid-β protein deposition and neurofibrillary tangles, hallmarks of Alzheimer’s disease (47). Hormones and cytokines secreted by adipose tissue can affect brain energy metabolism, neuronal survival, emotional states, and cognitive processes, and may cause neuroinflammation and neuronal damage (48–52). Research has linked obesity to gray matter atrophy in various brain regions, notably the prefrontal cortex, which is associated with schizophrenia and cognitive functioning (53, 54). Addressing obesity and its related metabolic effects may be important in managing cognitive impairments in individuals with schizophrenia.

Further correlation analysis revealed a negative association between working memory and BMI and TG, as well as a connection between visual learning and BMI and LDL-C in FEDN schizophrenia patients. Multiple linear regression analysis also revealed that a higher BMI predicted a decrease in working memory in FEDN schizophrenia patients. These findings underscore the potential harm of overweight/obesity on cognitive function in the early stage of schizophrenia, especially in specific cognitive domains such as executive function. However, due to limited relevant studies in FEDN schizophrenia patients, more research is needed to replicate our findings.

Additionally, the research results regarding the association between obesity and cognitive function in patients with schizophrenia who have received antipsychotic medication for a certain period are inconsistent. X. Guo et al. observed a significant association between higher BMI and lower scores on the visual reproduction and digit symbol tests (55). Likewise, S. Hidese et al. detected a negative correlation between BMI and the composite score of Brief Assessment of Cognition in Schizophrenia (BACS) (12). However, C. W. Wei et al.’s study revealed a positive correlation between BMI and language and visuospatial domains (14). Moreover, another study suggests that there is no association between obesity and cognitive impairment in individuals with schizophrenia (56). One speculation is that the more severe cognitive impairments in patients with schizophrenia may obscure the potential correlation between cognitive impairment and other risk factors. For instance, Rashid employed structural equation modeling to unveil the deleterious impact of obesity on the condition of patients with schizophrenia, thereby affecting cognition (15). Another speculation is that the association between obesity and cognition may be related to the ‘obesity paradox’ (57), where higher BMI in younger years is associated with decreased cognitive abilities, but higher BMI in later years is associated with improved cognition. Furthermore, these inconsistent results may also be attributed to the differences in assessment tools, disease progression, frequency of psychiatric episodes, use of antipsychotic medications, and family economic capacity. Therefore, more research is needed to explore the complex and dynamic relationship between obesity and cognitive function in patients with schizophrenia, especially in specific cognitive domains and different stages of the illness.

The strengths of this study lie in the examination of first-episode drug-naïve schizophrenia patients, and the absence of confounding factors, such as antipsychotic intervention and sex effects. However, this study had several limitations. First, due to its cross-sectional design, we were unable to track changes within each patient and determine causal relationships or moderating effects between variables. Therefore, future studies should focus on longitudinal studies of BMI changes in the same individual to more accurately assess the dynamic relationship between BMI and cognitive function, as well as their association with disease progression and treatment outcomes. We hope that such research will provide more evidence and guidance for cognitive rehabilitation in patients with FEDN schizophrenia. Second, our study solely focused on male schizophrenia patients, which limits generalization to the entire population with schizophrenia. To enhance the inclusivity and comprehensiveness of our observation, it is imperative to improve sample diversity by incorporating female patients and healthy individuals. Third, the sample size of this study was relatively small, which could increase the likelihood of obtaining false-negative results. Four, in this study, relying solely on BMI as a measure of obesity has limitations because it does not account for factors such as body fat percentage and differences in body shape. Future research could consider combining multiple methods to measure obesity, such as waist circumference and waist-to-height ratio (WHtR), and conducting more detailed subgroup analyses to comprehensively assess the relationship between the type and severity of obesity and schizophrenia. Finally, the possibility of selection bias cannot be ruled out, since our analysis only included subjects who were tested for all variables. Despite adjusting for various known confounders in our analyzes, there may still exist residual confounding.

## Conclusions

In conclusion, our findings indicate that the rate of overweight/obesity among FEDN schizophrenia patients was high. Additionally, overweight/obesity patients exhibited markedly inferior performance in working memory and visual learning compared to normal/under weight patients. Furthermore, regression analysis revealed that BMI significantly and negatively influenced working memory of FEDN schizophrenia patients. These results underscore the potential utility of markers for overweight/obesity as targets for both improvement and prevention strategies in addressing cognitive function in schizophrenia. This study provides more clues for clinical and scientific investigation of this intricate and multifaceted psychiatric disorder.

## Data availability statement

The original contributions presented in the study are included in the article/**Supplementary Material**. Further inquiries can be directed to the corresponding authors.

## Ethics statement

The studies involving humans were approved by the Medical Research Ethics Committee of the Affiliated Brain Hospital of Nanjing Medical University. The studies were conducted in accordance with the local legislation and institutional requirements. The participants provided their written informed consent to participate in this study. Written informed consent was obtained from the individual(s), and minor(s)’ legal guardian/next of kin, for the publication of any potentially identifiable images or data included in this article.

## Author contributions

XD: Data curation, Writing – original draft, Writing – review & editing, Conceptualization, Formal analysis, Methodology, Software. SL: Data curation, Writing – original draft, Writing – review & editing, Funding acquisition. YL: Data curation, Writing – review & editing. XF: Conceptualization, Methodology, Software, Writing – review & editing. RZ: Data curation, Investigation, Writing – review & editing. XS: Data curation, Writing – review & editing. JD: Funding acquisition, Project administration, Resources, Validation, Writing – review & editing. SX: Funding acquisition, Project administration, Resources, Validation, Writing – review & editing.
