# Supplementary material for: Association between increased BMI and cognitive function in first-episode drug-naïve male schizophrenia
Source: Front Psychiatry. 2024 Mar 5;15:1362674. doi: 10.3389/fpsyt.2024.1362674 (PMC10948420; doi:10.3389/fpsyt.2024.1362674)

Supplementary Material

The raw scores on 9 tests in the MCCB for both normal/under weight and overweight/obesity schizophrenia patients are displayed in Table S1. Additionally, the Pearson correlation heatmap among these raw scores and metabolic parameters is presented in Figure S1. The scatter plot of BMI and MCCB T-scores are shown in figure S2.

Table S1: raw scores on 9 tests in the MCCB in patients with normal/under weight and overweight/obesity.

|  | **Schizophrenia (n = 143)** | **groups** | | **t** | **P** |
| --- | --- | --- | --- | --- | --- |
|  |  | **normal/under weight (n = 95)** | **overweight/obesity (n = 48)** |  |  |
| TMT | 39.93 ± 14.78 | 40.59 ± 15.78 | 38.62 ± 12.65 | t=0.75 | 0.455 |
| BACS SC | 49.35 ± 11.93 | 49.98 ± 12.41 | 48.10 ± 10.92 | t=0.89 | 0.377 |
| HVLT-R | 22.83 ± 5.27 | 22.86 ± 5.15 | 22.75 ± 5.57 | t=0.12 | 0.904 |
| WMS-III SS | 14.76 ± 3.06 | 15.25 ± 3.22 | 13.81 ± 2.50 | t=2.70 | 0.008** |
| NAB Mazes | 15.43 ± 6.28 | 15.46 ± 6.37 | 15.35 ± 6.17 | t=0.10 | 0.922 |
| BVMT-R | 22.34 ± 6.94 | 23.35 ± 6.60 | 20.35 ± 7.24 | t=2.48 | 0.014* |
| Fluency | 21.72 ± 8.89 | 22.22 ± 10.08 | 20.73 ± 5.82 | t=0.95 | 0.345 |
| MSCEIT ME | 84.64 ± 15.91 | 84.47 ± 17.64 | 84.96 ± 11.87 | t=-0.17 | 0.866 |
| CPT-IP | 2.46 ± 4.00 | 2.71 ± 4.87 | 1.96 ± 0.69 | t=1.06 | 0.290 |

Means ± SD value, *p < 0.05, **p < 0.01, ***p < 0.001

Abbreviations: TMT, Trail Making Test: Part A; BACS SC, Brief Assessment of Cognition in Schizophrenia: Symbol Coding; HVLT-R, Hopkins Verbal Learning Test—Revised; WMS-III SS, Wechsler Memory Scale—Third Edition: Spatial Span; NAB Mazes, Neuropsychological Assessment Battery: Mazes; BVMT-R, Brief Visuospatial Memory Test—Revised (BVMT-R); Fluency, Category Fluency Test: Animal naming; MSCEIT ME, Mayer–Salovey–Caruso Emotional Intelligence Test: Managing Emotions; CPT-IP, Continuous Performance Test—Identical Pairs.

Figure S1: Pearson correlation heatmap among raw scores on 9 tests in the MCCB and metabolic parameters.


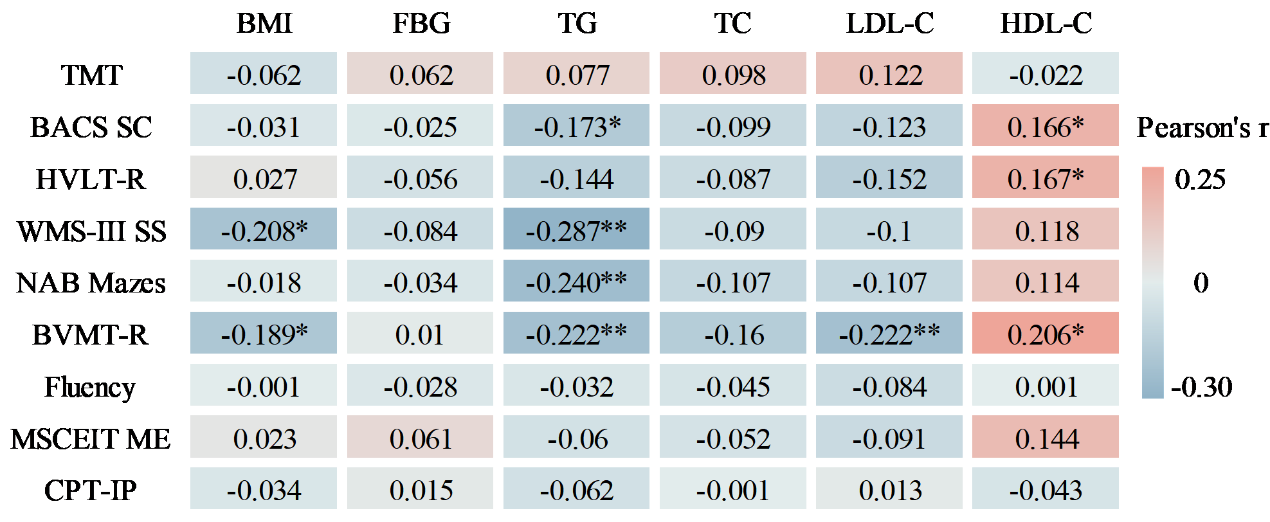


Abbreviations: TMT, Trail Making Test: Part A; BACS SC, Brief Assessment of Cognition in Schizophrenia: Symbol Coding; HVLT-R, Hopkins Verbal Learning Test—Revised; WMS-III SS, Wechsler Memory Scale—Third Edition: Spatial Span; NAB Mazes, Neuropsychological Assessment Battery: Mazes; BVMT-R, Brief Visuospatial Memory Test—Revised (BVMT-R); Fluency, Category Fluency Test: Animal naming; MSCEIT ME, Mayer–Salovey–Caruso Emotional Intelligence Test: Managing Emotions; CPT-IP, Continuous Performance Test—Identical Pairs.

Figure S2: The scatter plot of BMI and MCCB T-scores.


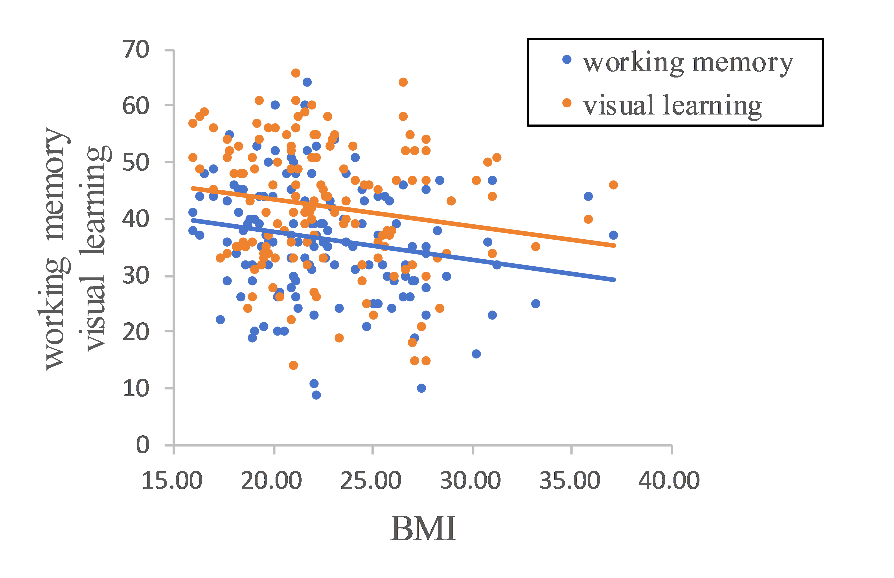

Supplement: Supplementary file 1 [file DataSheet_1.docx]
